# Supplementary material for: An Integrative Evaluation Method for the Biological Safety of Down and Feather Materials
Source: Int J Mol Sci. 2019 Mar 21;20(6):1434. doi: 10.3390/ijms20061434 (PMC6471580; doi:10.3390/ijms20061434)
Supplement: Supplementary file 1 [file ijms-20-01434-s001.pdf]

Int. J. Mol. Sci. **2019**, *20*, 1434; doi:10.3390/ijms20061434

Supplementary Materials

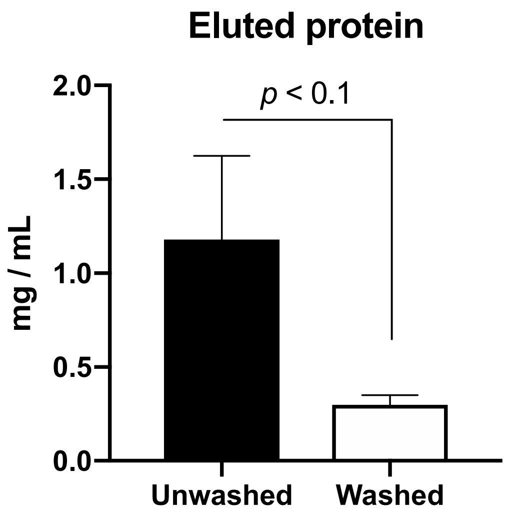

Figure S1. Protein contamination in extracts of down and feather samples; 0.1 g of down and feather samples were immersed in 10 mL sterile water and shaken for 1 min at room temperature. Then, the protein concentrations of the immersed water samples were quantified using Micro BCA Protein Assay (Thermo Fisher Scientific, Rockford, IL, USA) according to the manufacturer's protocol. n = 3, error bars indicate SE.

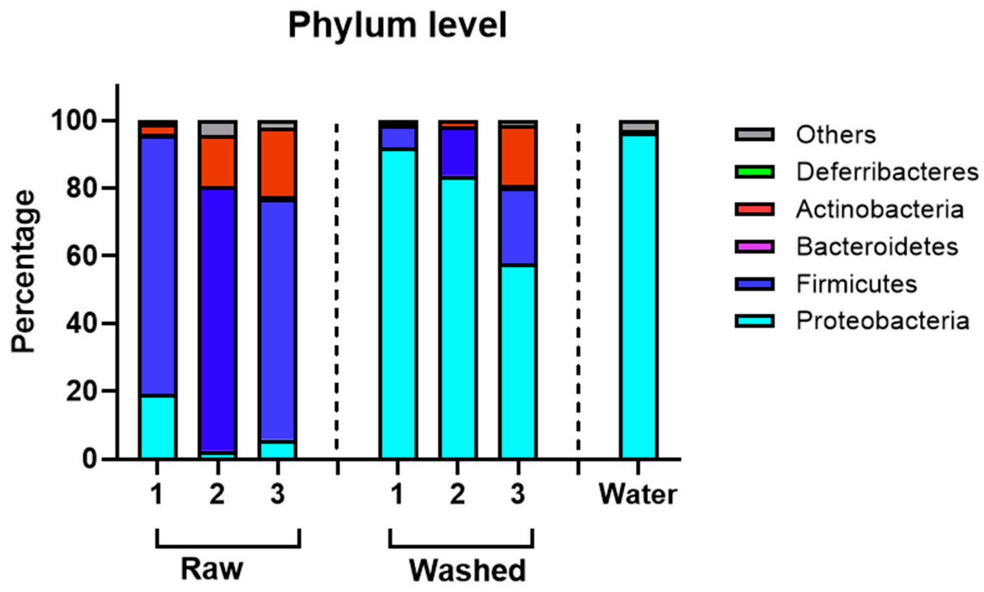

Figure S2. Phylum-level bacterial composition in down and feather samples.

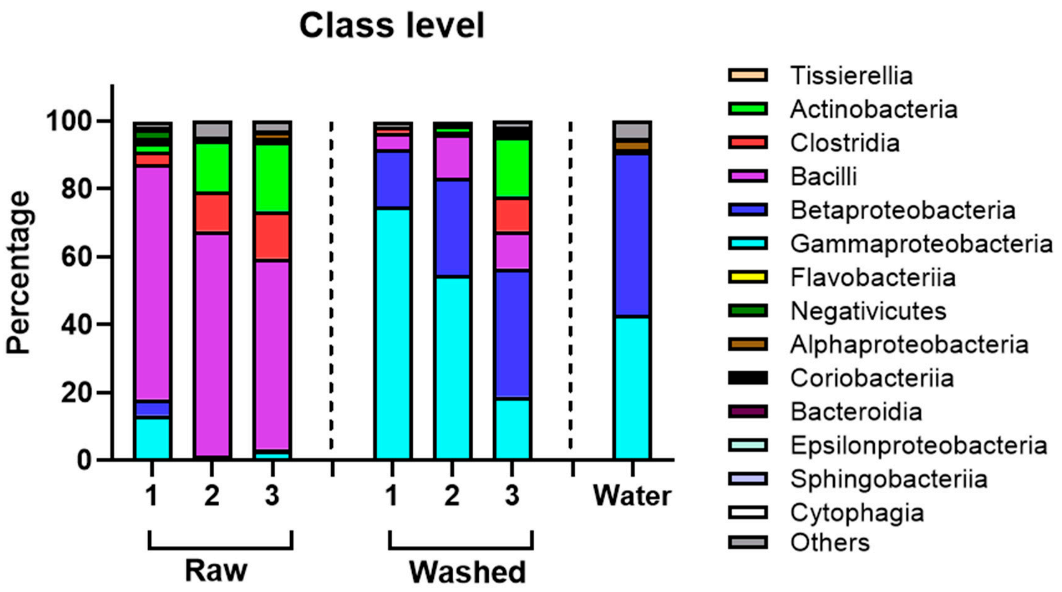

Figure S3. Class-level bacterial composition in down and feather samples.

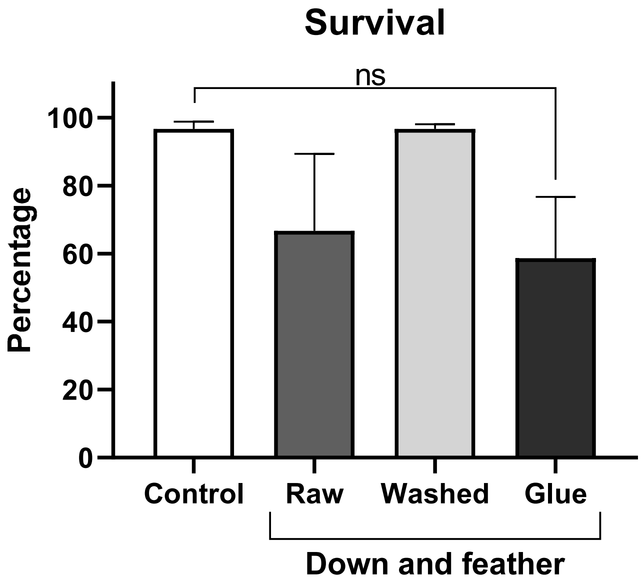

Figure S4. Survival rate of zebrafish embryos at 72 hours post fertilization (hpf). n = 4-6; each group contains 10 fish with triplicates, error bars indicate SD.

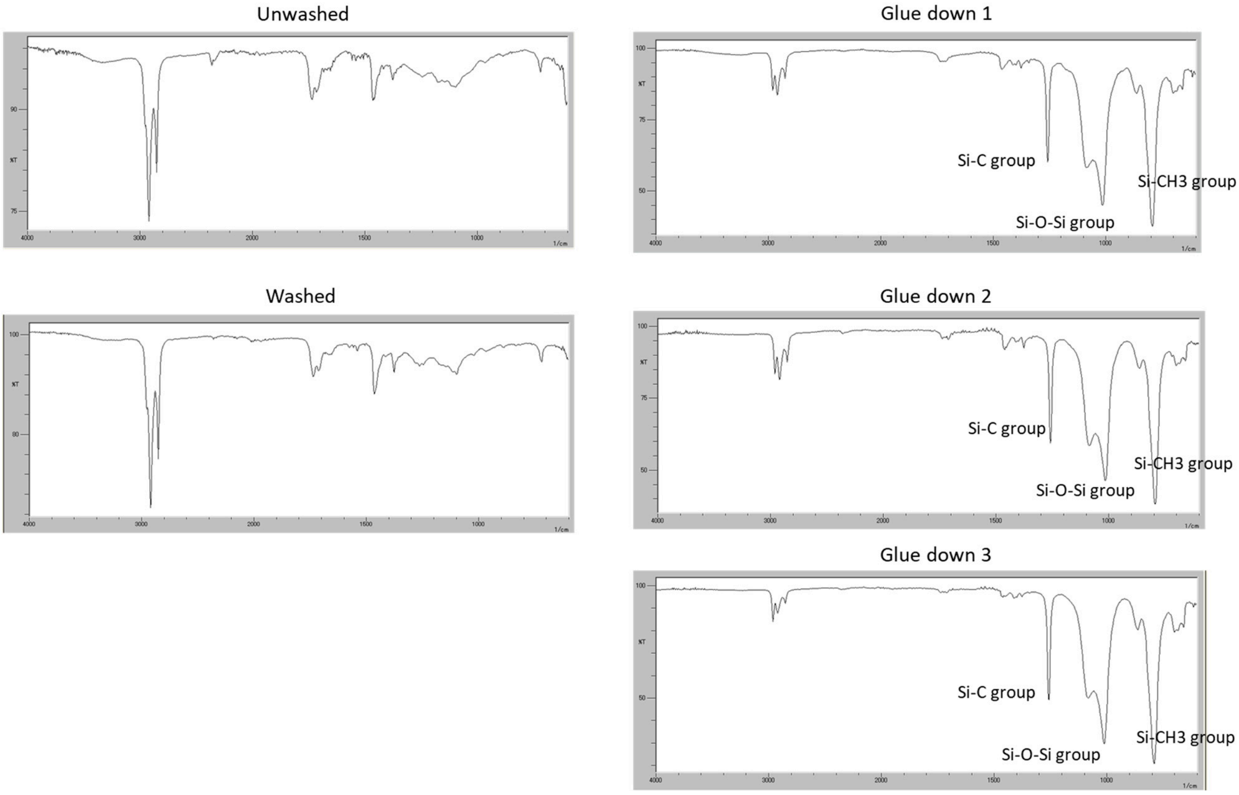

Figure S5. Silicon (Si) detection in unwashed, washed, and glue down and feather samples using Fourier transform infrared spectroscopy.

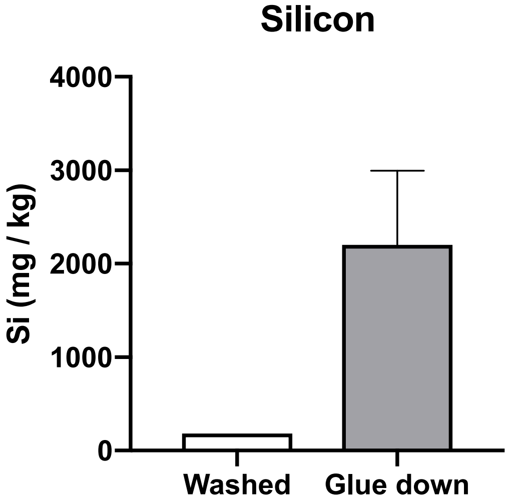

Figure S6. Silicon (Si) contamination in washed and glue down and feather samples. Si levels were measured by ICP-MS followed by microwave digestion. n = 1-3, error bars indicate SD.
